# Multifunctional gold nanostars for molecular imaging and cancer therapy

**DOI:** 10.3389/fchem.2015.00051

**Published:** 2015-08-12

**Authors:** Yang Liu, Hsiangkuo Yuan, Andrew M. Fales, Janna K. Register, Tuan Vo-Dinh

**Affiliations:** ^1^Fitzpatrick Institute for Photonics, Duke UniversityDurham, NC, USA; ^2^Department of Biomedical Engineering, Duke UniversityDurham, NC, USA; ^3^Department of Chemistry, Duke UniversityDurham, NC, USA

**Keywords:** multifunctional, plasmonics, gold nanostars, cancer imaging, cancer therapy

## Abstract

Plasmonics-active gold nanoparticles offer excellent potential in molecular imaging and cancer therapy. Among them, gold nanostars (AuNS) exhibit cross-platform flexibility as multimodal contrast agents for macroscopic X-ray computer tomography (CT), magnetic resonance imaging (MRI), positron emission tomography (PET), as well as nanoprobes for photoacoustic tomography (PAT), two-photon photoluminescence (TPL), and surface-enhanced Raman spectroscopy (SERS). Their surfactant-free surface enables versatile functionalization to enhance cancer targeting, and allow triggered drug release. AuNS can also be used as an efficient platform for drug carrying, photothermal therapy, and photodynamic therapy (PDT). This review paper presents the latest progress regarding AuNS as a promising nanoplatform for cancer nanotheranostics. Future research directions with AuNS for biomedical applications will also be discussed.

## Introduction

Nanotheranostics, combining diagnostic and therapeutic functions into one nanoparticle, has attracted great attention due to its promise as a powerful tool for personalized therapy to treat cancer, which contributes to more than seven million deaths each year (Ferlay et al., 2010; Jemal et al., 2010; Wang et al., 2014). Theranostic nanoprobes can potentially be used for image-guided cancer therapy with improved targeting specificity and therapeutic efficacy (Choi et al., 2012). Nanoparticles of sizes ranging from 20 to 100 nm accumulate in tumors through the well-known enhanced permeability and retention (EPR) effect that originates from the leaky tumor vasculature (Maeda, 2001; Maeda et al., 2001, 2003; Sykes et al., 2014). Furthermore, targeting ligands, including antibodies and peptides, can be functionalized on nanoparticles for active cancer targeting (Kim et al., 2011b). Various nanoplatforms, including liposomes, iron oxide nanoparticles, and gold nanoparticles have been developed for cancer imaging and treatment (Ahmad et al., 2010; Xie et al., 2010; Lee et al., 2014; Wang et al., 2014). In particular, gold nanoparticles have demonstrated great biocompatibility, plasmonic properties, and convenience in versatile functionalization through strong gold-thiol interactions (30–40 kcal/mol), as can be exemplified by gold nanorods, nanoshells, and nanocages in preclinical and clinical cancer management (Ramachandran et al., 2003; Loo et al., 2004; Alekseeva et al., 2006; Choi et al., 2012; Huschka et al., 2012; Xia et al., 2012b; Ahmadi and Arami, 2014; Lee et al., 2014; Xia and Xia, 2014). Those established plasmonic gold nanoplatforms (nanorods, nanoshells and nanocages) have been widely used in previous studies for *in vitro* and *in vivo* investigations of cancer imaging with positron emission tomography (PET), magnetic resonance imaging (MRI), X-ray computer tomography (CT), high resolution optical imaging with labeled fluorescence dyes as well as photothermal therapy (PTT) and photodynamic therapy (PDT). Furthermore, gold nanospheres and nanoshells have been used in clinical trials for drug delivery and photothermal therapy, respectively (Gad et al., 2012). Therefore, novel nanoplatforms are of great interest for cancer detection and treatment.

**Figure 1:**
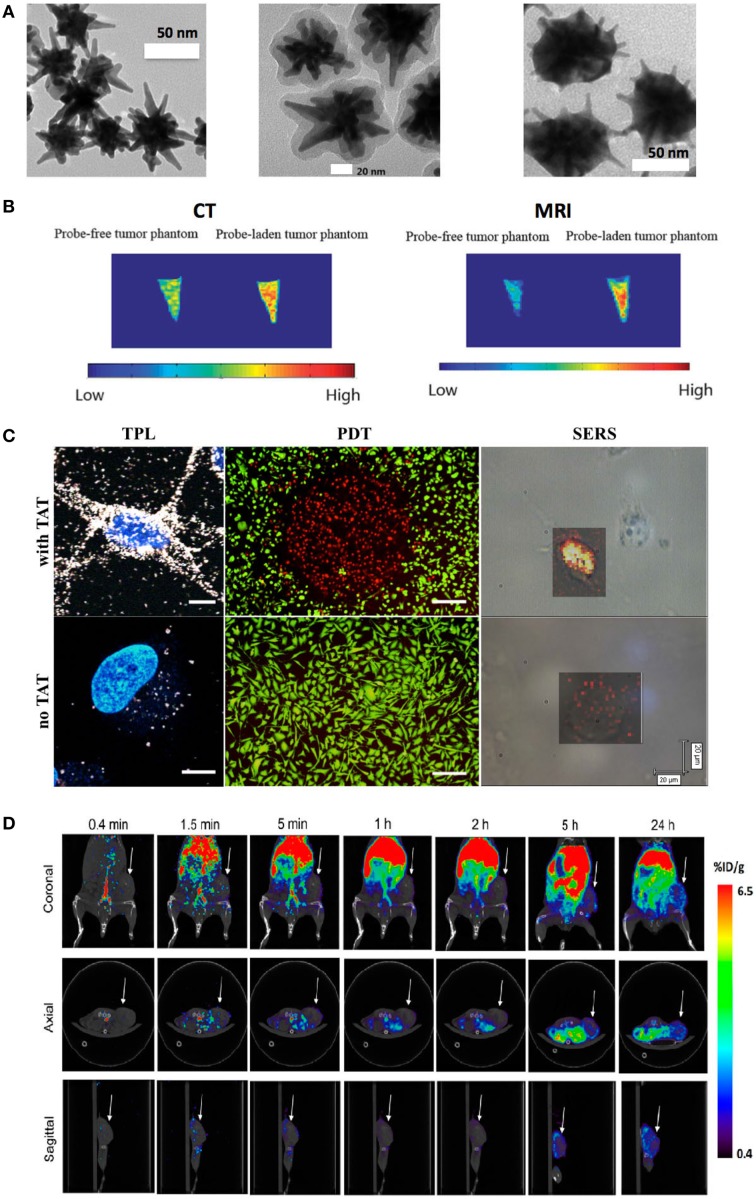
**(A)** TEM images of synthesized AuNS (left), silica-coated AuNS (middle), and silver coated-AuNS (right). **(B)** CT (left) and MRI (right) imaging with developed multifunctional AuNS nanoprobe. **(C)** Demonstration of TAT-peptide enhanced TPL imaging, photodynamic therapy (PDT), and surface-enhanced Raman scattering (SERS) imaging after 1-h incubation with the AuNS probe. Scale bars for TPL, PDT and SERS image are 10, 250, and 20 μm, respectively. **(D)** PET/CT imaging with ^64^Cu labeled AuNS probe, which was injected intravenously through tail vein. Dynamic imaging at various time pointes were acquired. Arrow shows tumor (Fales et al., 2011, 2013, 2014; Yuan et al., 2013a; Liu et al., 2015b).

Gold nanostars (AuNS), with multiple sharp branches (Figure 1A), have superior tip-enhanced plasmonic properties in the near-infrared (NIR) tissue optical window, which is suitable for *in vivo* biomedical applications. Plasmonic AuNS have been applied for *in vivo* lymphatic system mapping with photoacoustic tomography (PAT) (Kim et al., 2011a). AuNS with silica shells have been found to be internalized into living cells and can be used for intracellular imaging (Rodriguez-Lorenzo et al., 2011; Fales et al., 2013; Yuan et al., 2013a). In addition, AuNS surface-enhanced Raman scattering (SERS) nanoprobes have been applied for immuno-SERS microscopy of the tumor suppressor p63, imaged in prostate biopsies (Schutz et al., 2011). SERS takes advantage of a unique phenomenon on certain metal nanoparticles, surface plasmon resonance (SPR). Surface plasmon refers to oscillating electrons within the conduction band when the metallic nanostructure surface is excited by an external electromagnetic field. The oscillating electrons can generate a secondary electromagnetic field, which is added to the external electromagnetic field to result in SPR. Incident photon energy, when in resonance with the surface plasmon, magnifies the local electromagnetic field that dramatically enhances the intrinsically weak Raman signal. The SERS enhancement factor is typically 10^6^–10^8^-fold and can be up to 10^15^-fold in hot spots, where the electromagnetic field is extremely intense (Liu et al., 2015b). By combining resonance enhancement, surface-enhanced resonance Raman scattering (SERRS) shows even greater Raman signal enhancement than SERS alone. Silica-coated AuNS SERRS nanoprobes have been used for visualizing brain tumor margins and microscopic tumor invasion (Harmsen et al., 2015). Our group has developed a novel toxic surfactant-free AuNS synthesis method that greatly improves the biocompatibility and the versatility of surface functionalization (Yuan et al., 2012b). In this review paper, we will focus on the latest progress achieved in our laboratory related to AuNS and discuss their bright future for theranostic applications.

## Multimodal imaging

AuNS provide a powerful tool for 3D *in vivo* tracking and disease detection with whole body scans. For CT imaging, gold nanoparticles are superior to traditional iodinated contrast agents, since gold has a higher atomic number (*Z* = 79) and k-edge value (80.7 keV). In addition, the X-ray attenuation of iodine is decreased by water while that of gold is not. Gold nanoparticles can absorb X-ray, which subsequently generate photoelectric and Compton effects. These effects lead to formation of secondary electrons and reactive oxygen species (ROS), the yield of which correlates with the surface area of nanoparticles (Misawa and Takahashi, 2011). Since star-shaped geometry exhibits greater surface area than the spherical counterpart of equivalent size, AuNS can be an improved sensitizer for radiation therapy. For MRI imaging, Gd^3+^ ions have been linked to the AuNS surface through 1,4,7,10-tetraazacyclododecane-1,4,7,10-tetraacetic acid (DOTA) chelator. As shown in Figure 1B, tumor cells loaded with multifunctional AuNS probes display high intensity under CT and MRI examination (Liu et al., 2013a). PET scan provides an extremely sensitive 3D imaging method. The sensitivity could reach picomolar for PET compared to micromolar for MRI. We performed PET imaging with ^64^Cu-labeled AuNS nanoprobes for dynamic imaging up to 24 h (Figure 1D). *In vivo* tracking results showed that the developed AuNS nanoprobes accumulate gradually in tumor with 3.3:1 tumor-to-muscle ratio at the end of 24 h (Liu et al., 2015b). These studies exemplify the potential of AuNS for whole body imaging.

In addition, AuNS have been exploited as a powerful optical contrast agent under multiphoton microscope for high-resolution imaging. With an exceptionally high two-photon photoluminescence (TPL) [more than one million Göeppert-Mayer (GM) two-photon action cross-section (TPACS)], AuNS offer superior signal contrast than quantum dots or other organic fluorophores for multiphoton optical imaging (Yuan et al., 2012b). Furthermore, a recent study investigated TPL of single gold nanoparticle with different shapes and the TPACS were reported to be 83,500, 1.5 × 10^3^, 4.2 × 10^4^, 4.0 × 10^6^ GM for nanosphere, nanocube, nanotriangle, nanorod and nanostar, respectively (Gao et al., 2014). The TPACS of AuNS is almost two orders higher than that of gold nanorod, which is the highest one among other shapes. With an intense TPL emission, AuNS can be used not only for real-time *in vivo* tracking, but also for sensitive tumor detection following systemic injection of AuNS (Yuan et al., 2012c). As shown from TPL imaging in Figure 1C, transactivator of transcription (TAT)-functionalized AuNS nanoprobes have much stronger signal in cells than that of AuNS without TAT functionalization. TAT is a well-known cell-penetrating peptide with capability to increase nanoparticle cellular uptake. Although the TPL imaging depth is limited by the extent of laser penetration, optical microscopy offers the highest spatial resolution among most animal imaging modalities. AuNS are also suitable to be used under PAT with high extinction coefficient (~10^10^ M^−1^cm^−1^) (Xia et al., 2012a; Yuan et al., 2014). Being a potent contrast agent for PAT, which is an optical-ultrasound hybrid imaging technology, the particokinetics and biodistribution of the AuNS can be studied with deeper imaging depth and larger field of view.

Furthermore, AuNS have been applied for SERS detection with tip-enhanced plasmonics. The reported SERS enhancement factor is several orders higher than that of gold nanospheres (Yuan et al., 2013a). SERS applies nanometallic structures to enhance “fingerprint” Raman spectra, which provides a method for molecular sensing with high sensitivity (Liu and Sun, 2011; Liu et al., 2013b,c; Yuan et al., 2013b; Zhao et al., 2014a). Our group demonstrated the first analytical application of SERS in chemical analysis using nanostructured metal substrates 30 years ago, and has been working on various types of SERS nanoplatforms, including nanogratings, nanorod arrays, nanowires and AuNS, for biochemical sensing (Meier et al., 1985; Enlow et al., 1986; Alak and Vo-Dinh, 1987; Bello et al., 1989; Vo-Dinh et al., 1994, 2013; Stokes et al., 2004; Yuan et al., 2013a). SERRS AuNS nanoprobes have been developed to perform quantitative *ex vivo* multiplex detection with high sensitivity (Yuan et al., 2013b). The AuNS nanoprobes have also been used for SERS imaging for cancer cells as shown in Figure 1C (Fales et al., 2013). SERS images were acquired at 633 nm with a 2-μm step size by using a Renishaw Raman microscope. The 633 nm resonant dye, DTDC was used to label the AuNS for SERS detection. SERS imaging shows that TAT-functionalized AuNS nanoprobes have much stronger signal inside cells. Recently, we have reported the synthesis of silver-coated AuNS (AuNS@Ag) that provide over an order of magnitude increase in SERS signal when compared to AuNS alone (Fales et al., 2014). We have applied the AuNS@Ag for *in vitro* homogenous nucleic acid detection, which demonstrated AuNS' strong capability for molecular sensing with the SERS method (Wang et al., 2015). With such flexibility, AuNS thus are a promising nanoplatform for multimodality imaging from whole body scan to high-resolution optical imaging as well as molecular sensing.

## Brain tumor imaging

The potential of AuNS as an efficient contrast agent has further been investigated in animal brain tumor models (Yuan et al., 2014). Through cranial window chambers implanted with tumor cells in live animals, AuNS have been employed for angiography with high spatial resolution; cerebral capillaries were clearly visible with minimal tissue autofluorescence background. Unlike conventional contrast agents (e.g., FITC-dextran) showing signal decay within 30 min, AuNS can be coated with polyethylene glycol (PEG) to achieve a much longer intravascular signal stability due to its extended serum half-life (several hours) and lower degree of extravasation.

The main challenge of tumor imaging is delivering the contrast sufficiently to the target. Several physiological barriers exist between the injection site and the target tumor cells. For example, nanoparticles need to survive immunoclearance, extravasate tumor vessels, permeate the blood brain barrier (BBB), diffuse through interstitium, and penetrate the plasma membrane into cells (Chrastina et al., 2011; Goldberg et al., 2013). For brain tumor imaging, the greatest challenge lies in the permeation of the BBB. Tight junctions between the endothelial cells and podocytes from the astrocytes form a highly selective barrier that prevents large molecules from passing through. To date, the typical 24-h post-injection brain accumulation of nanoparticles may still be less than 0.1% of the initial dosage (Khlebtsov and Dykman, 2011); this is similar to those obtained after monoclonal antibody infusion. Several nanoparticle-based platforms have been studied, including transferrin-containing gold nanoparticles (Wiley et al., 2013), polysorbate 80-coated poly(n-butyl cyanoacrylate) dextran polymers (Koffie et al., 2011), and angiopep2 peptide-functionalized dendrimers (Yan et al., 2012). Since AuNS are a strong contrast agent, it is therefore prudent to investigate the effect of surface functionalization on brain tumor targeting.

Nanoparticles accumulate in tumors typically through passive and active mechanisms. While surface PEGylation achieves the former, the latter requires surface functionalization of peptides or proteins. Upon systemic exposure, PEGylated AuNS accumulate passively near the tumor periphery and around blood vessels. Hyper-neovascularity along the tumor edge and interstitial fluid pressure gradient at the boundary attenuate the penetration of AuNS deep into the tumor. PEG-AuNS also accumulate in vascular endothelial cells but with minimal true transcytosis(Ye et al., 2013), where paracellular extravasation is most likely attributed to the defective tight junction at the tumor site. With extravasation depending on the nanoparticle size and incubation time (Yuan et al., 1994; Popovic et al., 2010), smaller nanoparticles or longer incubation may further increase the tumor accumulation or extravasation depth.

**Figure 2:**
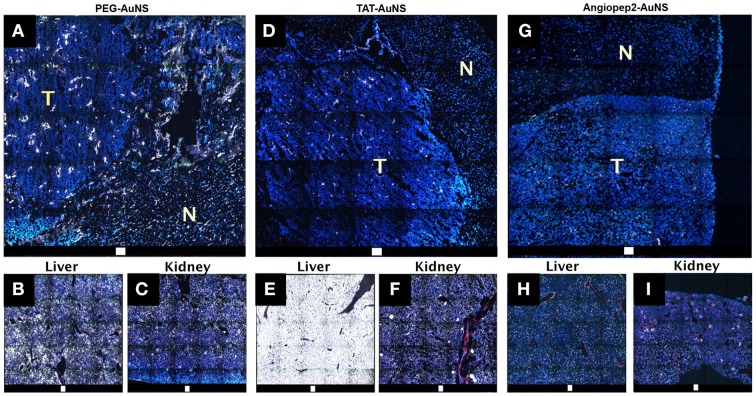
**TPL images of DAPI- (nucleus; blue) and CD31 (blood vessel; red)-stained cryosectioned specimens from perfused brain excised 48-hr after injection (5 pmole; 80 nm) of neutral PEG-AuNS (A–C) and positively-charged TAT-AuNS (D–F), and injection (1 pmole; 50 nm) of angiopep2-AuNS (G–I)**. Large-area tile images showing distribution of PEG-AuNS **(A)**, TAT-AuNS **(D)**, Angiopep2-AuNS **(G)** preferentially in the brain tumor than normal brain. T, tumor. N, normal. Charged TAT-AuNS accumulated greater in the liver hence lower intratumoral accumulation. Smaller angiopep2-AuNS was more widespread in the tumor. Scale bar: 100 μm (Yuan et al., 2014).

Comparing the brain tumor AuNS delivery through passive and active mechanisms, we coated 80-nm AuNS with PEG (PEG-AuNS) and TAT peptides (TAT-AuNS), and 50-nm AuNS with angiopep2 peptides (angiopep2-AuNS). Angiopep2-peptide has recently been exploited to deliver nano-drugs into the brain *via* lipoprotein receptor related protein (Gabathuler, 2010). PEG-AuNS accumulated in the tumor periphery and parenchyma but much less in the normal brain. They also accumulated inside endothelial cells and perivascular spaces, particularly in the tumor regions of defective vascular integrity. As shown in Figure 2, TAT-AuNS accumulated less in tumor but extensively in liver compared to PEG-AuNS. Positively charged TAT possibly attracted opsonins, causing greater entrapment in the reticuloendothelial system. In contrast, smaller angiopep2-AuNS had a deeper distribution inside tumor parenchyma with relatively less liver accumulation. Calculated from the AuNS intensity ratio from the images collected under the same microscopic settings, the average tumor/liver AuNS density ratios are 0.32, 0.03 and 1.2 for PEG-, TAT- and angiopep2-coated AuNS. These findings suggest that enhanced tumor BBB permeation with selected intratumoral delivery can be achieved with proper control of AuNS sizes and selection of surface ligand chemistry.

## AuNS for phototherapy

AuNS have also been applied for PDT and photothermal therapy (PTT). PDT uses a photosensitizer that generates ROS upon irradiation to kill cells, while photothermal therapy transduces light to heat for cancer hyperthermia or ablation. The effect of laser type on PTT has been investigated before and the results showed that pulsed lasers could use 10 times less energy to kill cancer cells than that used for continuous-wavelength (CW) lasers (Huang and El-Sayed, 2011). That is because pulsed-laser with gold nanoparticles can lead to a variety of therapeutic effects including protein denaturation, cell cavitation, bubble formation from shock waves and plasma generation (Pustovalov et al., 2008). With the nanoparticle's tumor targeting effect and focused irradiation to the tumor site, phototherapy can achieve greater tumor therapeutic specificity with less off-target effects. The therapeutic potentials have been demonstrated on gold nanoshells, nanocages, and nanorods (Von Maltzahn et al., 2009; Xie et al., 2010; Day et al., 2011). Gold nanoshells were used to perform PTT (800-nm diode laser, 4 W/cm^2^ intensity for 3 min.) with murine xenograft models and results showed significant survival rate improvement for the treatment group with 57% of mice remaining tumor free at the end of 90 days, while the mean survival date for the control group was only 13.3 days. Gold nanocages functionalized with HER2-antibody have been used to perform PTT with a femtosecond Ti:Sapphire laser (810 nm, 1.5 W/cm^2^ for 5 min) for SK-BR-3 breast cancer cells. The treatment group shows a majority of cells died while there was little cellular death in the control group (Skrabalak et al., 2007). Gold nanorods with T790 dye embedded in a silica shell were developed to perform PDT and the improved treatment efficiency has been demonstrated with HepG2 cancer cells (Zhao et al., 2014b). In particular, the star-shape geometry of AuNS generates a strong tip-enhanced plasmonic effect that greatly increases the photothermal transduction efficiency especially in the NIR tissue optical window. Successful photothermolysis has been demonstrated in both single photon and two photon settings (Yuan et al., 2012a,c). An ultralow irradiance of 0.2 W/cm^2^ (at using 850 nm pulsed laser), which was below the maximal permissible exposure of skin, was reported. A recent study demonstrated that AuNS exhibit higher photothermal transduction efficiency than gold nanoshells (90–94% for AuNS compared to 61% for gold nanoshells). *In vivo* experiment also revealed a successful photothermal ablation of aggressive sarcoma tumor in mouse after AuNS systemical injection through tail vein (Liu et al., 2015a). In addition, we also loaded Protoporphyrin IX onto silica-coated AuNS for PDT showing specific cytotoxic effect only on the UV exposed region (Figure 1C) (Fales et al., 2013). These results demonstrated that AuNS have great potential for PDT and photothermal therapy in future preclinical cancer treatment studies.

## Conclusion and future perspective

The AuNS synthesized with the surfactant-free method provide a novel nanoplatform for cancer nanotheranostics. The multifunctional AuNS nanoprobes can be used for future pre-treatment cancer diagnostics with PET, MRI, and CT scan, intraoperative imaging with TPL, PAT, and SERS, and image-guided therapy with PDT and PTT. The plasmonic AuNS is a promising theranostic nanoplatform since they have combined capabilities for diagnostics with various imaging modalities from whole-body to subcellular level, therapeutics with phototherapy and drug delivery, and surface functionalization versatility for cancer targeting. Compared to other gold plasmonic nanoplatforms, the AuNS nanoprobes have unique advantages of tip-enhanced plasmonics, high TPL cross-section, and excellent photon-to-heat conversion efficiency, toxic surfactant-free synthesis. Further studies on relationship between AuNS properties including particle size and surface charge, and their biodistribution are still required to improve nanoprobe uptake in tumor for better biomedical applications. With promising findings from previous studies, AuNS demonstrate great potential for translational medicine studies as a combined nanoplatform for both cancer imaging and therapy.

### Conflict of interest statement

The authors declare that the research was conducted in the absence of any commercial or financial relationships that could be construed as a potential conflict of interest.
